# Revealing the Influence of Zn Content on the Microstructure and Mechanical Properties of Bimodal Mg-Zn-Gd-Sm Alloy

**DOI:** 10.3390/ma18184226

**Published:** 2025-09-09

**Authors:** Hansong Xue, Zengjun Wei, Shanyi Lan, Yang Zhou, Ming Zhang, Jun Li, Ying Liu, Jia She, Jia Hu, Bin Jiang

**Affiliations:** 1 College of Materials Science and Engineering, Chongqing University, Chongqing 400044, China; zjwei1218@163.com (Z.W.); lanshanyi31@163.com (S.L.); claudezhou0206@163.com (Y.Z.); 202309021081@stu.cqu.edu.cn (M.Z.); lijcqu@163.com (J.L.); yingliu0821@163.com (Y.L.); jiashe@cqu.edu.cn (J.S.); hujia827@cqu.edu.cn (J.H.); jiangbinrong@cqu.edu.cn (B.J.); 2 National Engineering Research Center for Magnesium Alloys, Chongqing University, Chongqing 400044, China

**Keywords:** Mg-Zn-RE series magnesium alloy, dynamic recrystallization, bimodal-grained structure, mechanical properties

## Abstract

The development of low-cost and high-performance Mg alloys is an important way to achieve further application of magnesium alloys. In this work, the as-extruded Mg_98.3−x_Zn_x_Gd_1_Sm_0.7_ alloy with excellent mechanical properties is successfully prepared by regulating the bimodal-grained structure. The effect of the Zn content on the microstructure evolution and mechanical properties of the as-extruded Mg_98.3−x_Zn_x_Gd_1_Sm_0.7_ alloy is systematically investigated. The results show that the addition of Zn increases the dynamic recrystallization (DRX) fraction and weakens the basal texture of the as-extruded alloy. The Mg_98.05_Zn_0.25_Gd_1_Sm_0.7_ alloy exhibits a typical bimodal-grained structure. A large amount of geometrically necessary dislocations (GNDs) are generated at the interface between the soft zone and the hard zone of the bimodal-grained structure during the plastic deformation process, resulting in back stress strengthening, thereby improving the strength of the alloy. And it achieves exceptional mechanical properties with an ultimate tensile strength (UTS) of 330 MPa, a yield strength (YS) of 248 MPa, and an elongation (EL) of 18.5% at room temperature. This paper provides a new idea for introducing a heterogeneous structure and improving the strength of low-cost Mg alloys.

## 1. Introduction

Magnesium (Mg) alloys, the lightest metal structural material in engineering application, are widely used in aerospace, automotive, and electronics industries owing to their exceptional specific strength, specific stiffness, and damping capacity [1,2]. However, the low absolute strength and high alloy cost constitute the primary limitations restricting the broader application of Mg alloys. Therefore, advancing the development of low-cost and high-strength Mg alloys has become one of the research hotspots in recent years [3].

At present, Mg-Zn-RE alloys have significant advantages in many aspects, which can meet the needs of industries [4,5,6,7]. For instance, Li et al. [8] systematically investigated the precipitate evolution and strengthening mechanisms of Mg-9Gd-3Y-xZn-0.5Zr alloys. And the alloy achieved optimal mechanical properties with an ultimate tensile strength (UTS) of 330 MPa, a yield strength (YS) of 287.3 MPa, and an elongation (EL) of 5.0% due to the synergistic strengthening effect of multiple precipitates. Zhang et al. [9] demonstrated that a strong fiber texture appeared in the Mg-Zn-Nd-Zr-Yb alloy through rapid solidification ribbon consolidation (RSRC). This deformation process resulted in remarkable grain boundary (GB) strengthening and texture strengthening effects, leading to significantly enhanced mechanical properties with a YS of 400 MPa and a UTS of 407 MPa. However, current research on high-strength Mg-Zn-RE alloys faces two key challenges: firstly, the rare earth (RE) content is usually high (>15 wt.%), driving up production costs; secondly, the alloys exhibit a low elongation and a significant strength–ductility trade-off. The present study is focused on a Mg–Zn–Gd–Sm alloy. The Gd element has a high solid solubility in Mg, which can play a prominent solid solution strengthening effect. And the addition of Gd can significantly weaken the strong basal texture in the Mg alloy and improve the plastic deformation ability of the alloy [10]. The Sm element exhibits the highest solid solubility (5.8 wt.%) in Mg among light rare earth elements (LREEs), providing a strong strengthening effect at a much lower cost than heavy rare earth elements (HREEs) [11].

The introduction of a bimodal-grained structure is an important way to strengthen Mg-Zn-RE alloys. A certain proportion of coarse grains (CGs) and fine grains (FGs) can make Mg alloys have both high strength and high ductility [12]. This is mainly due to the strain gradient between the CGs and FGs. In the process of plastic deformation, a high density of geometrically necessary dislocations is generated at the interface, inducing back stress strengthening in the process of coordinating plastic deformation [13]. The methods of introducing a bimodal-grained structure include controlling the solidification process, severe plastic deformation (SPD), heat treatment, and so on [5]. Nakata et al. [14] produced a bimodal-grained structure in a Mg-Zn-Ca-Mn alloy through rolling with reheating at 450 °C. They found that the alloy’s texture was significantly weakened due to the non-uniform distribution of dislocations. Gao et al. [15] fabricated a ZN20 alloy with a bimodal-grained structure by equal channel angular pressing (ECAP) and extrusion. The alloy’s ductility could be increased by 40% compared with the as-extruded alloy, which was without a bimodal-grained structure. However, the complex formation process of such a heterogeneous structure (bimodal-grained structure) hinders their industrial-scale production, significantly limiting the widespread application of heterogeneous-structured Mg alloys. To address this challenge, some scholars have proposed that regulating the morphology and distribution of the second phase in the alloy can promote the formation of the bimodal-grained structure during the deformation process [16,17,18]. Until now, studies like regulating the morphology and distribution of the second phase to introduce a bimodal-grained structure have rarely been reported. Our research goal is to regulate the morphology and distribution of the second phase, thereby tailoring the dynamic recrystallization (DRX) behavior to form a heterogeneous structure (bimodal-grained structure).

In this study, a bimodal Mg-Zn-Gd-Sm alloy was successfully fabricated by Zn content regulation and conventional hot extrusion. The Mg_98.05_Zn_0.25_Gd_1_Sm_0.7_ alloy exhibits a high UTS of 330 MPa, a YS of 248 MPa, and good elongation of 18.5%. The effect of the Zn content on the microstructure of the Mg-Zn-Gd-Sm alloy, especially the formation of the bimodal microstructure, was systematically studied. The strengthening effect and the strengthening mechanism of microstructure evolution on the mechanical properties of the Mg-Zn-Gd-Sm alloy were further investigated. This study can provide some guiding significance for the development of Mg alloy strengthening and toughening research.

## 2. Materials and Methods

This paper presents four alloys with different contents of Mg_98.3−x_Zn_x_Gd_1_Sm_0.7_ (x = 0.25, 0.5, 0.75, 1 at.%) alloys. According to the alloy composition, pure Mg (99.99 wt.%), pure Zn (99.99 wt.%), Mg-25Gd (wt.%) master alloy, and Mg-20Sm (wt.%) master alloy were used as raw materials. The melting operation was conducted in a crucible resistance furnace with CO_2_ + SF_6_ (volume ratio 99:1) as the protective atmosphere at 720–750 °C. Then, the melted alloy was put into a heat treatment furnace and subjected to homogenization annealing at 500 °C for 12 h to reduce element segregation. The annealed alloy was processed into a Φ80 mm cylindrical billet. The billet was preheated at 450 °C for 2 h and then extruded at 450 °C with an extrusion ratio of 16:1.

The chemical composition of the alloy was analyzed using an X-ray Fluorescence Spectrometer (XRF-1800CCDE, SHIMADZU, Kyoto, Japan). The specific composition of the alloy is detailed in Table 1. The metallographic structure of the alloy was observed by an inverted optical microscope (OM, XJP-6A, COIC, Beijing, China). The phase composition of the alloy was analyzed by an X-ray diffraction analyzer (XRD, D/MAX-2500PC, Rigaku, Tokyo, Japan). The analysis of the second-phase composition and morphology was performed using scanning electron microscopy (SEM, TESCAN VEGA Ⅲ LMU, Tescan, Brno, Czech Republic) and an energy-dispersive spectrometer (EDS, OXFORD INCA Energy 350, Oxford Instruments, Abingdon, UK). The grain orientation and texture in the micro area were analyzed by electron backscattered diffraction (EBSD, JEOL JSM-7800F, JEOL, Tokyo, Japan), and the data processing software was Azteccrystal 2.1. The mechanical properties were tested by a microelectronic universal testing machine (CMT-5105, 100KN, MTS, Shanghai, China).

## 3. Results and Discussion

### 3.1. Microstructure of the As-Cast Alloys

**Figure 1:**
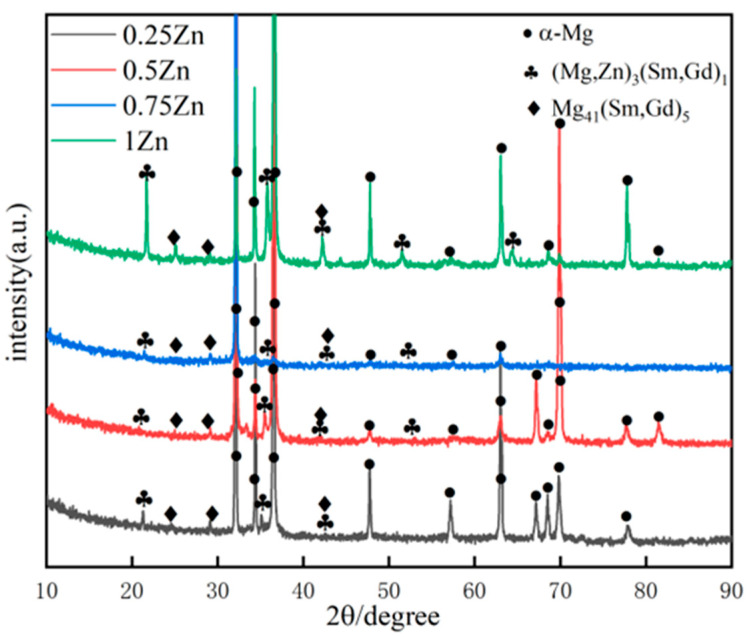
XRD patterns of as-cast Mg_98.3−x_Zn_x_Gd_1_Sm_0.7_ alloys with varying Zn contents.

Figure 1 presents the XRD patterns of as-cast Mg_98.3−x_Zn_x_Gd_1_Sm_0.7_ (x = 0.25, 0.5, 0.75, 1 at.%) alloys with varying Zn contents, indicating that the as-cast alloys consist of α-Mg, (Mg,Zn)_3_(Sm,Gd)_1_, and Mg_41_(Sm,Gd)_5_. But the diffraction peak intensity of these phases is low. This phenomenon can be attributed to the limited variety and content of alloying elements, which restricts second-phase formation. Furthermore, the water-cooling process employed during casting, characterized by its rapid cooling rate, resulted in a fraction of Zn, Gd, and Sm elements remaining dissolved in the Mg matrix without precipitation.

**Figure 2:**
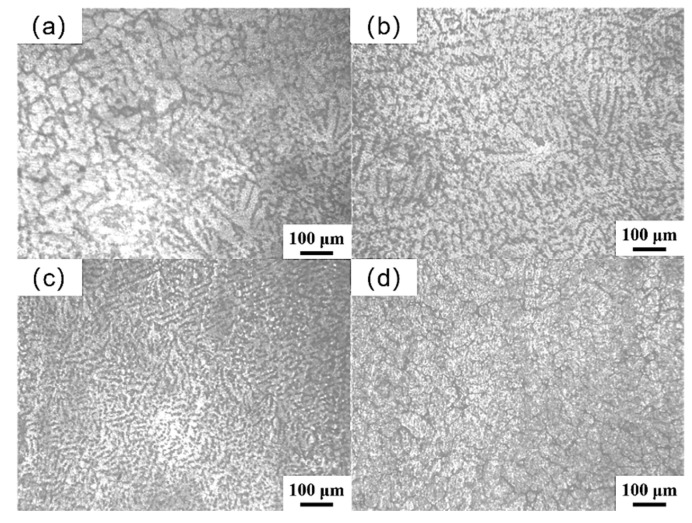
Microstructure observed by optical microscopy of as-cast Mg_98.3−x_Zn_x_Gd_1_Sm_0.7_ alloys with varying Zn contents: (**a**) 0.25% Zn; (**b**) 0.5% Zn; (**c**) 0.75% Zn; (**d**) 1% Zn.

Figure 2 shows the microstructure evolution of Mg_98.3−x_Zn_x_Gd_1_Sm_0.7_ (x = 0.25, 0.5, 0.75, 1 at.%) alloys with a varying Zn content. As depicted in Figure 2a, the alloy comprises α-Mg dendritic structures and co-distributed eutectic compounds. At 0.5 at.% Zn addition, the microstructure of the alloy is refined, though the effect is not pronounced. As the Zn content increases to 0.75 at.%, the coarse α-Mg dendrites are obviously refined. However, further increasing Zn to 1 at.%, the dendrites are coarsened.

**Figure 3:**
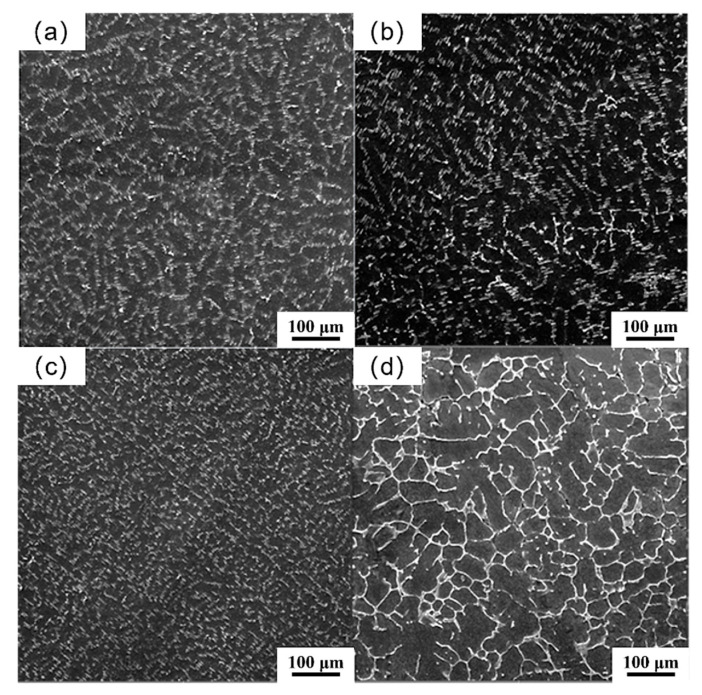
SEM morphology (low magnification) of as-cast Mg_98.3−x_Zn_x_Gd_1_Sm_0.7_ alloys with varying Zn contents: (**a**) 0.25% Zn; (**b**) 0.5% Zn; (**c**) 0.75% Zn; (**d**) 1% Zn.

Figure 3 shows the low-magnification SEM morphology of the alloys. It can be seen that the second phase in the as-cast alloy is mainly located in the interdendritic regions. When 0.25 at.%, 0.5 at.%, and 0.75 at.% Zn are added, the second phase of the as-cast alloy exhibits a fine needle-like morphology distributed along grain boundaries, as shown in Figure 3a–c. When 1 at.% Zn is added, the second phase of the as-cast alloy exhibits a semi-continuous mesh structure, as shown in Figure 3d.

**Figure 4:**
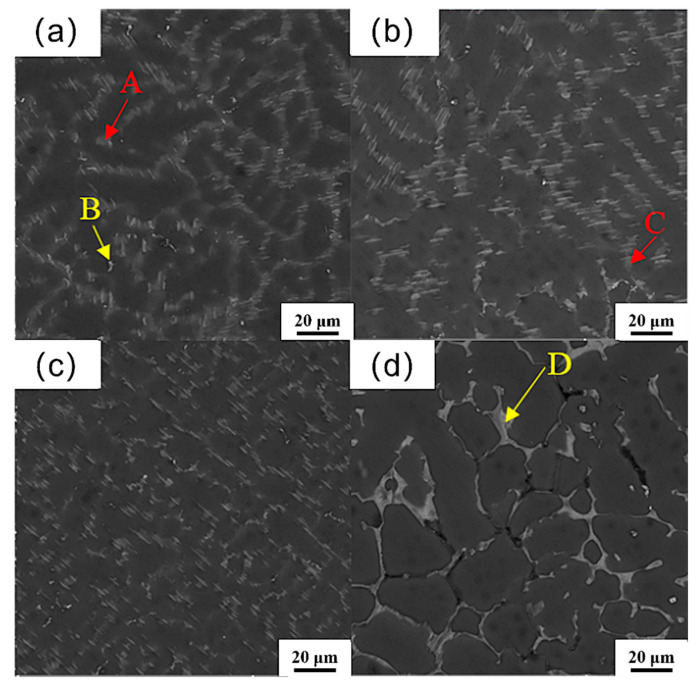
SEM morphology (high magnification) of the as-cast Mg_98.3−x_Zn_x_Gd_1_Sm_0.7_ alloys with varying Zn contents: (**a**) 0.25% Zn; (**b**) 0.5% Zn; (**c**) 0.75% Zn; (**d**) 1% Zn.

Figure 4 shows the high-magnification SEM morphology of the alloy. And the quantitative analysis by EDS is shown in Table 2. Since the EDS analysis is inherently semiquantitative, the type of the second phase needs to be determined by combining XRD and EDS analysis. According to the analysis of EDS results, the gray-white phase (points A and C) is mainly composed of Mg, Gd, and Sm elements, and the ratio of Mg / (Gd + Sm) is close to 41/5. Combined with the XRD spectrum, the analysis indicates that the phase is Mg_41_(Sm,Gd)_5_, which exhibits fine needle-like morphology distributed along grain boundaries (Figure 4a,b). The bright-white phase (points B and D) includes Mg, Gd, Zn, and Sm elements. It can be combined with XRD to determine that it is a (Mg,Zn)_3_(Sm,Gd)_1_ phase (Figure 4a,d). The reason for the transformation of the second phase from a fine needle-like to semi-continuous mesh structure can be that with the increase in the Zn element content in the alloy, more Sm and Gd elements are consumed to form the (Mg,Zn)_3_(Sm,Gd)_1_ phase, and the Mg_41_(Sm,Gd)_5_ phase decreases.

In addition, SEM morphology analysis reveals that the grain size of the alloy initially decreases and subsequently increases with the increase in Zn content. According to the growth restriction factor (GRF) theory of solute atoms [19], the addition of the Zn element leads to an increase in the GRF value. The elevated GRF value induces significant compositional undercooling at the solid–liquid interface front during alloy solidification. Meanwhile, the enhanced undercooling promotes nucleation, thereby resulting in grain refinement. However, when the Zn content reaches 1 at.%, the extensively formed secondary phases release latent heat of crystallization, which reduces the undercooling at the solid–liquid interface front. Consequently, the nucleation rate decreases, and the grain growth inhibition effect weakens [20].

### 3.2. Microstructure of As-Extruded Alloys

**Figure 5:**
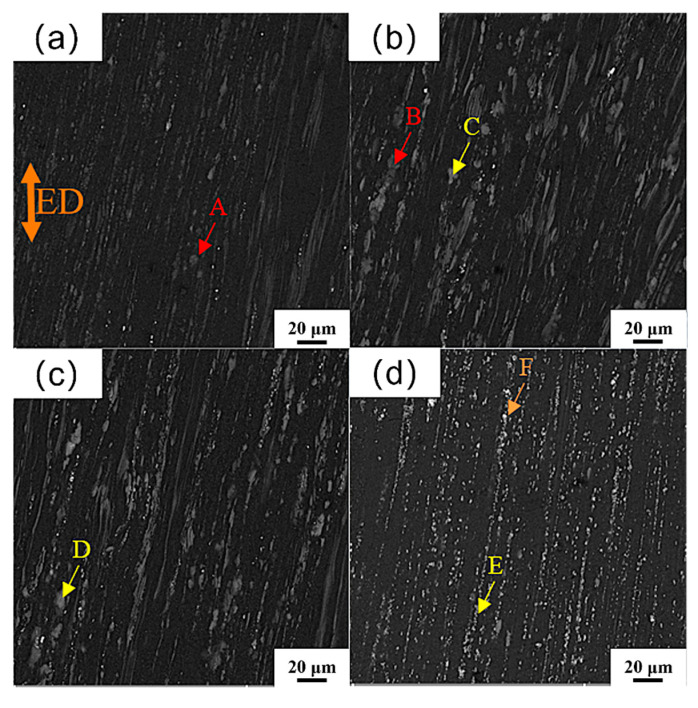
SEM morphology of as-extruded Mg_98.3-x_Zn_x_Gd_1_Sm_0.7_ alloys with varying Zn contents: (**a**) 0.25% Zn; (**b**) 0.5% Zn; (**c**) 0.75% Zn; (**d**) 1% Zn.

Figure 5 shows the SEM morphology of the as-extruded Mg_98.3−x_Zn_x_Gd_1_Sm_0.7_ (x = 0.25, 0.5, 0.75, 1 at.%) alloy parallel to the extrusion direction (ED). The second phase in the as-extruded alloy is significantly broken and refined through hot extrusion, forming pronounced extrusion flow lines along the ED due to the extrusion force. The quantitative analysis by EDS is shown in Table 3. Combined with the XRD spectrum, it can be concluded that the coarse light-gray second phase (points A and B) is Mg_41_(Sm,Gd)_5_. The tiny gray-white second-phase particles (points C, D, E) obviously distributed along the ED are (Mg,Zn)_3_(Sm,Gd)_1_, and the number of the second-phase particles increases with the increase in Zn content. And some bright-white particles (points F) are RE-rich phases. The absence of the RE-rich phase in the XRD patterns may be attributed to their relatively low content below the detection limit.

In order to further investigate the effect of the Zn content on the microstructure of as-extruded Mg_98.3−x_Zn_x_Gd_1_Sm_0.7_ (x = 0.25, 0.5, 0.75, 1 at.%) alloys, the as-extruded alloys were characterized by EBSD.

**Figure 6:**
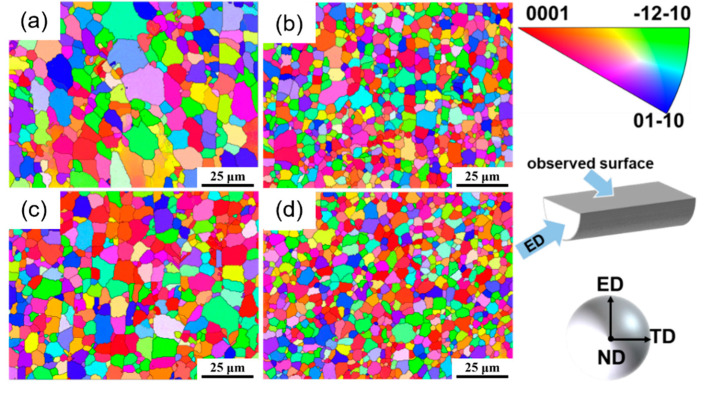
Inverse pole figure (IPF) maps of as-extruded Mg_98.3−x_Zn_x_Gd_1_Sm_0.7_ alloys with varying Zn contents: (**a**) 0.25% Zn; (**b**) 0.5% Zn; (**c**) 0.75% Zn; (**d**) 1% Zn.

**Figure 7:**
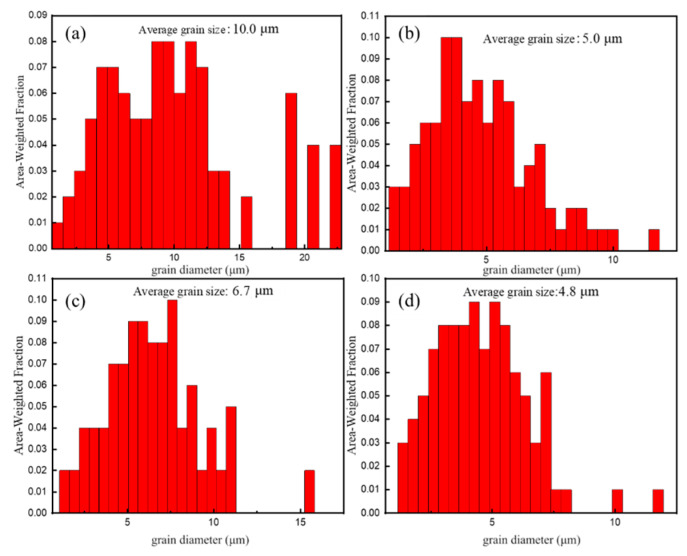
Grain size distribution maps of as-extruded Mg_98.3−x_Zn_x_Gd_1_Sm_0.7_ alloys with varying Zn contents: (**a**) 0.25% Zn; (**b**) 0.5% Zn; (**c**) 0.75% Zn; (**d**) 1% Zn.

Figure 6 shows the inverse pole figure (IPF) maps of the as-extruded alloys, while Figure 7 shows the grain size distribution of the as-extruded alloys. It can be seen that the grain sizes of the four alloys are quite different after hot extrusion, and the average grain sizes are 10 μm, 5 μm, 6.7 μm, and 4.8 μm, respectively. Among them, the grain size distribution of the as-extruded Mg_98.3−x_Zn_x_Gd_1_Sm_0.7_ (x = 0.5, 0.75, 1 at.%) alloys is relatively uniform, while the grain size of the as-extruded Mg_98.05_Zn_0.25_Gd_1_Sm_0.7_ alloy is non-uniform, exhibiting a mixture of CGs and FGs. According to the previous exploration [21], this structure with a non-uniform grain size is a typical bimodal-grained structure. The phenomenon is attributed to the low Zn content, which leads to inadequate second-phase particles for effective grain boundary pinning, thereby failing to prevent recrystallized grain growth and ultimately resulting in a bimodal-grained structure in the as-extruded Mg_98.05_Zn_0.25_Gd_1_Sm_0.7_ alloy [22]. However, the number of (Mg,Zn)_3_(Sm,Gd)_1_ phase particles increase in the alloy with the increase in Zn content. According to the particle stimulated nucleation (PSN) mechanism, broken fine second-phase particles can serve as nucleation sites forDRX, thereby increasing the nucleation rate [22]. In addition, a large number of dispersed second-phase particles can effectively hinder the growth of DRX grains to a certain extent, thus further refining the recrystallized grains [23]. As a result, with an increasing Zn content, the grain size of the alloy is gradually refined and the distribution is gradually uniform.

**Figure 8:**
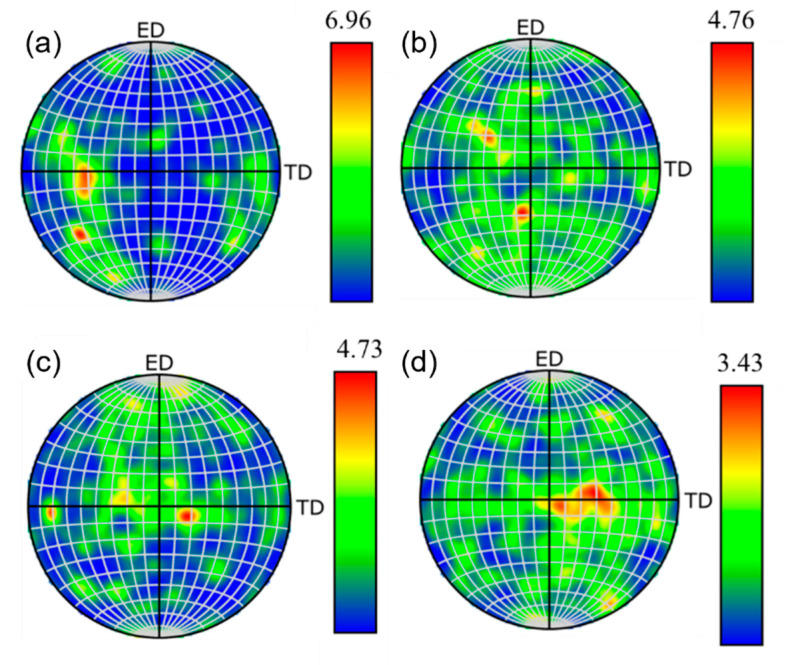
(0001) pole figures of as-extruded Mg_98.3−x_Zn_x_Gd_1_Sm_0.7_ alloys with varying Zn contents: (**a**) 0.25% Zn; (**b**) 0.5% Zn; (**c**) 0.75% Zn; (**d**) 1% Zn.

Figure 8 shows the pole figures of the as-extruded alloys with varying Zn contents. All four alloys exhibit typical basal textures, while the texture intensity shows a decreasing trend with an increasing Zn addition, quantified as 6.96, 4.76, 4.73, and 3.43, respectively. This texture weakening can be attributed to two mechanisms: (1) The solid solution of Zn reduces the axial ratio (c/a) and increases the stacking fault energy (SFE) of the Mg matrix, which enhances prismatic slip activity and consequently improves ductility while weakening texture intensity [24]. (2) Due to the PSN mechanism in the hot extrusion process, a large number of broken fine second phases can promote the DRX nucleation, and can effectively pin the grain boundary, which can effectively weaken the texture [25]. As 0.25 at.% Zn is added, the alloy exhibits a maximum pole density of 6.96. This relatively high texture intensity results from inadequate second-phase particles, leading to weak PSN effects during extrusion. The grain boundary cannot be effectively pinned, leading to recrystallized grain growth and consequently a higher texture strength. As the Zn content increases, the progressively enhanced PSN effect leads to texture weakening, and the ductility of the alloy is improved.

**Figure 9:**
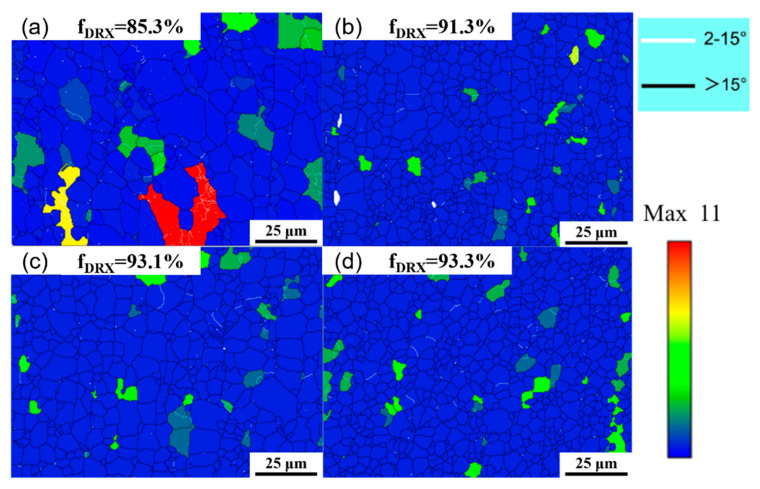
Grain orientation spread (GOS) maps of extruded Mg_98.3−x_Zn_x_Gd_1_Sm_0.7_ alloys with varying Zn contents: (**a**) 0.25% Zn; (**b**) 0.5% Zn; (**c**) 0.75% Zn; (**d**) 1% Zn.

Recrystallization during hot extrusion can be called dynamic recrystallization [26,27]. Figure 9 shows the grain orientation spread (GOS) diagram of the as-extruded Mg_98.3−x_Zn_x_Gd_1_Sm_0.7_ (x = 0.25, 0.5, 0.75, 1 at.%) alloy. The blue area in the diagram is the grain with a GOS below 1°, which is identified as recrystallized grains. The other color is the grain with a GOS above 1°, which is identified as unrecrystallized grains. According to the GOS results, the recrystallization fraction of the as-extruded Mg_98.3−x_Zn_x_Gd_1_Sm_0.7_ (x = 0.25, 0.5, 0.75, 1 at.%) alloys is 85.3%, 91.3%, 93.1%, and 93.3%, respectively. All four as-extruded alloys exhibit high recrystallization fractions, which results from a high extrusion temperature. With the increase in Zn content, the recrystallization fraction of the alloy gradually increases. This phenomenon originates from the fine dispersed (Mg,Zn)_3_(Sm,Gd) phase particles that increase with the increasing Zn content; this increases the particle–matrix interfacial contact area and promotes PSN effects, thereby increasing nucleation sites for DRX.

As shown in Figure 9, low-angle grain boundaries (LAGBs, <15°) are primarily concentrated within grain interiors, while high-angle grain boundaries (HAGBs, ≥15°) primarily concentrated around the grains. The four alloys exhibit relatively low fractions of LAGBs, and with the increase in Zn content, the fractions of LAGBs decrease from 12.6% to 4.12%. This phenomenon occurs because LAGBs continuously absorb dislocations during recrystallization, finally transforming into HAGBs. Additionally, the PSN effect from fine-dispersed second-phase particles enhances recrystallized grain nucleation. The newly formed HAGBs further serve as nucleation sites for DRX, significantly improving DRX efficiency [28].

**Figure 10:**
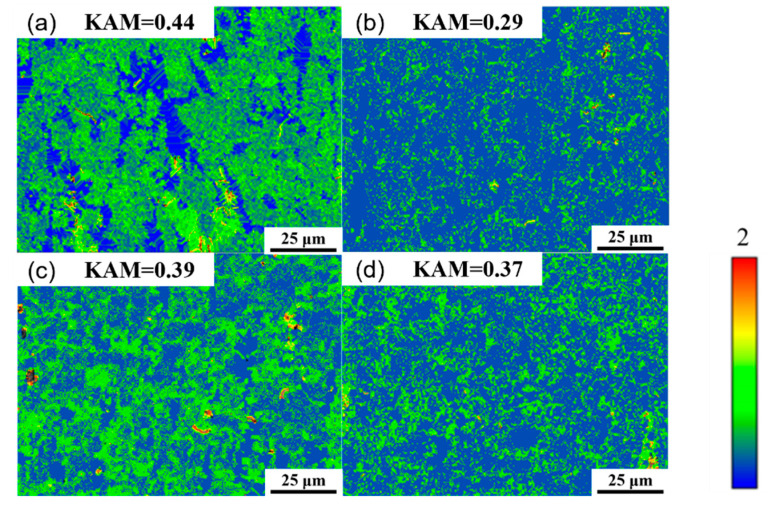
Kernel average misorientation (KAM) maps of as-extruded Mg_98.3−x_Zn_x_Gd_1_Sm_0.7_ alloys with varying Zn contents: (**a**) 0.25% Zn; (**b**) 0.5% Zn; (**c**) 0.75% Zn; (**d**) 1% Zn.

Figure 10 shows the Kernel average misorientation (KAM) of the as-extruded Mg_98.3−x_Zn_x_Gd_1_Sm_0.7_ (x = 0.25, 0.5, 0.75, 1 at.%) alloy. The KAM value effectively characterizes intra-granular orientation gradient variations within the alloy and reflect the magnitude of localized plastic strain induced by dislocation slip. The KAM values of four alloys are 0.44, 0.29, 0.39, and 0.37, respectively. The four alloys exhibit low KAM values, indicating that the internal dislocation density of the four alloys is low [29]. At 0.25 at.% Zn, the highest KAM value (0.44) reflects the maximal dislocation density and localized plastic strain.

**Figure 11:**
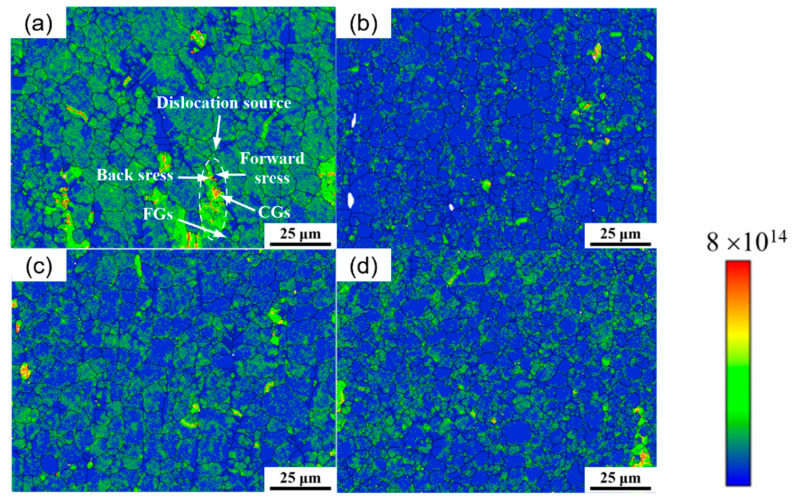
Geometrically necessary dislocation (GND) maps of as-extruded Mg_98.3−x_Zn_x_Gd_1_Sm_0.7_ alloys with different Zn contents: (**a**) 0.25% Zn; (**b**) 0.5% Zn; (**c**) 0.75% Zn; (**d**) 1% Zn.

Figure 11 shows the geometrically necessary dislocation (GND) map of as-extruded Mg_98.3−x_Zn_x_Gd_1_Sm_0.7_ (x = 0.25, 0.5, 0.75, 1 at.%) alloys. As shown in Figure 11a, the bimodal-grained structure exhibits the highest GND density, predominantly localized at heterogeneous structure boundaries. During the initial plastic deformation stage, CGs (soft zones) preferentially accommodate plastic strain, while FGs (hard zones) maintain elastic deformation. With progressive deformation, this uncoordinated deformation induces a continuously escalating strain gradient within the alloy. At the time, a large number of GNDs will appear in the material to coordinate the deformation behavior of the material. Therefore, high-density GNDs are first generated in the FGs region, while a large number of GNDs are accumulated in the CGs region only near the heterogeneous interface, which indicates that there is an obvious strain gradient between the heterogeneous structures [30]. And the back stress is generated in the soft zone (CGs zone), and the forward stress is generated in the hard zone (FGs zone). In the FGs region, the interaction between the grain boundary and dislocation is beneficial to generate stress concentration near the grain boundary, so as to activate the non-basal slip of fine grain and increase the plasticity of the alloy [31]. When the alloy is plastically deformed, the stress increases continuously, and the interaction between the GND and the movable dislocation occurs continuously. The dislocation movement is hindered, and the strength of the alloy is also greatly improved. The grain size distribution of the Mg_98.05_Zn_0.25_Gd_1_Sm_0.7_ alloy is the most non-uniform, and the difference between the soft zone and the hard zone is the most obvious. Different grain boundary strengthening causes the interaction between heterogeneous layers [30].

**Figure 12:**
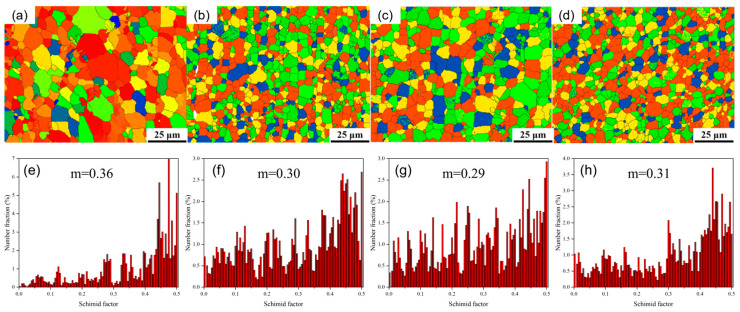
Quantitative analysis of slip Schmidt factor (SF) distribution maps of as-extruded Mg_98.3−x_Zn_x_Gd_1_Sm_0.7_ alloys with varying Zn contents along the ED direction (0001) base plane: (**a**,**e**) 0.25% Zn; (**b**,**f**) 0.5% Zn; (**c**,**g**) 0.75% Zn; (**d**,**h**) 1% Zn.

Figure 12 shows the Schmid factor (SF) distribution maps corresponding to the basal (0001) <11−20> slip in the as-extruded Mg_98.3−x_Zn_x_Gd_1_Sm_0.7_ (x = 0.25, 0.5, 0.75, 1 at.%) alloy. As quantified in Figure 12a–d, the average SF values for the basal slip in these alloys are 0.36, 0.30, 0.29, and 0.31, respectively. All four alloys exhibit relatively high average SF values, indicating that the basal slip systems are more readily activated compared to other Mg alloy compositions, with enhanced basal slip activity. So, they are expected to demonstrate superior plastic deformability.

### 3.3. Mechanical Properties of As-Extruded Alloys

**Figure 13:**
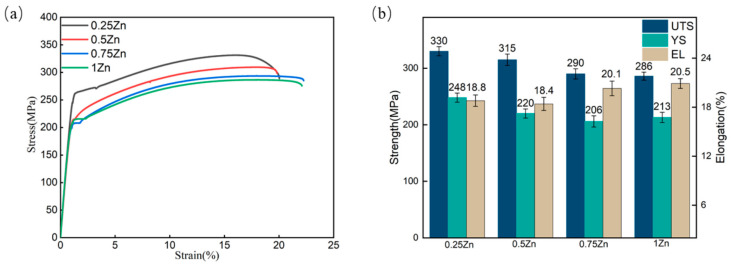
The tensile mechanical properties of as-extruded Mg_98.3−x_Zn_x_Gd_1_Sm_0.7_ alloys with varying Zn contents. (**a**) Tensile engineering stress-strain curve; (**b**) Mechanical properties histogram.

Figure 13 presents the room-temperature tensile mechanical properties of as-extruded Mg_98.3−x_Zn_x_Gd_1_Sm_0.7_ (x = 0.25, 0.5, 0.75, 1 at.%) alloys. With an increasing Zn content, the alloy exhibits a gradual deterioration in comprehensive mechanical properties. The Mg_98.05_Zn_0.25_Gd_1_Sm_0.7_ alloy achieves optimal properties: a UTS of 330 MPa, a YS of 248 MPa, and an EL of 18.8%. The (Mg,Zn)_3_(Sm,Gd)_1_ eutectic phase in the Mg_98.05_Zn_0.25_Gd_1_Sm_0.7_ alloy is lower, and the weak PSN effect is generated during the deformation process of the alloy. The alloy exhibits a low recrystallization fraction and strong basal texture. After the deformation of the alloy, the grain size distribution is obviously non-uniform. The interaction between the soft zone and the hard zone causes the alloy to produce a large number of GNDs during plastic deformation to compensate for the strain gradient generated by deformation, which results in the back stress in the soft zone and the forward stress in the hard zone. The coupling effect of the two is manifested as macroscopic back stress strengthening, which further improves the strength of the alloy. With the continuous addition of the Zn element, the grains of the alloy gradually tend to be uniform, and the difference between the soft zone and the hard zone gradually decreases. The GNDs generated during the deformation of the alloy gradually decrease, the obstacles to dislocation movement gradually decrease, and the strength of the alloy gradually decreases. But the ductility of the alloy is improved under the combined action of the sufficient DRX and high Schmid factor [32].

When the alloy is plastically deformed, the stress increases continuously, and the interaction between the GND and the movable dislocation occurs continuously. The dislocation movement is hindered, and the strength of the alloy is also greatly improved [30]. The grain size distribution of the Mg_98.05_Zn_0.25_Gd_1_Sm_0.7_ alloy is the most non-uniform, and the difference between the soft zone and the hard zone is the most obvious. With the continuous addition of the Zn element, the grains of the alloy gradually tend to be uniform, and the difference between the soft zone and the hard zone gradually decreases. The GNDs generated during the deformation of the alloy gradually decrease, the obstacles to dislocation movement gradually decrease, and the strength of the alloy gradually decreases [33].

In general, the Mg_98.05_Zn_0.25_Gd_1_Sm_0.7_ alloy exhibits good comprehensive mechanical properties due to the comprehensive influence of grain size, texture strength, DRX degree, dislocation density, and other factors.

## 4. Conclusions

A novel bimodal Mg_98.3−x_Zn_x_Gd_1_Sm_0.7_ (x = 0.25, 0.5, 0.75, 1 at.%) alloy with good strength–ductility synergy was developed by the conventional extrusion process, coupled with regulating the morphology and distribution of the second phase. The formation mechanism and strengthening effect of the bimodal-grained structure in the Mg_98.3−x_Zn_x_Gd_1_Sm_0.7_ alloy were investigated. The main conclusions are as follows:

(1) The bimodal-grained microstructure is successfully introduced in the Mg_98.05_Zn_0.25_Gd_1_Sm_0.7_ alloy. The formation mechanism of the bimodal-grained structure is that the broken fine second-phase particles act as nucleation sites for DRX, enhancing the nucleation rate. Moreover, the dispersed second-phase particles inhibit DRX grain growth, contributing to further grain refinement. However, inadequate second-phase particles limit the PSN effect, leading to the growth of recrystallized grains and eventually forming the bimodal-grained structure.

(2) In the bimodal-grained structure, a large amount of GNDs are generated at the interface between the soft zone (CGs zone) and the hard zone (FGs zone), promoting the back stress in the soft zone and the forward stress in the hard zone. The coupling effect of the two is manifested as macroscopic back stress strengthening, which further improves the strength of the alloy. Thus, the as-extruded Mg_98.05_Zn_0.25_Gd_1_Sm_0.7_ alloy exhibits the optimal comprehensive mechanical properties, and the UTS, YS, and EL are 330 MPa, 248 MPa, and 18.8%, respectively, at RT.

(3) The improvement in alloy strength is attributed to back stress strengthening and texture strengthening. The as-extruded alloys exhibit relatively high average SF values, indicating that the basal slip systems are more readily activated, with enhanced basal slip activity, which could improve the ductility.

## Figures and Tables

**Table 1:** The specific composition of Mg_98.3−x_Zn_x_Gd_1_Sm_0.7_ alloys.

Alloy Composition (at.%)	Alloy Composition (wt.%)	Zn (wt.%)	Gd (wt.%)	Sm (wt.%)	Mg (wt.%)
Mg_98.05_Zn_0.25_Gd_1_Sm_0.7_	Mg-0.6Zn-6Gd-1.7Sm	0.9	7.2	2.1	Bal. (Balance)
Mg_97.8_Zn_0.5_Gd_1_Sm_0.7_	Mg-1.2Zn-6Gd-1.7Sm	1.5	7.4	2.2	Bal.
Mg_97.55_Zn_0.75_Gd_1_Sm_0.7_	Mg-1.9Zn-6Gd-1.7Sm	2.1	7.0	1.7	Bal.
Mg_97.3_Zn_1_Gd_1_Sm_0.7_	Mg-2.5Zn-6Gd-1.7Sm	2.8	6.7	2.3	Bal.

**Table 2:** EDS results of each point in Figure 4 (at.%).

Position	A	B	C	D
Mg	87.59	75.52	93.48	72.21
Zn	-	9.48	-	12.43
Sm	2.12	3.72	1.40	4.54
Gd	10.29	11.28	5.12	10.82

**Table 3:** EDS results of each point in Figure 5 (at.%).

Points	A	B	C	D	E	F
Mg	83.55	85.59	75.95	78.93	78.02	60.39
Zn	-	-	12.94	11.90	12.61	-
Sm	3.16	4.12	3.57	2.78	3.15	9.96
Gd	13.29	10.29	7.54	6.39	6.22	29.65

## Data Availability

The original contributions presented in this study are included in the article. Further inquiries can be directed to the corresponding author.
